# Targeting Pain and Depression in Alzheimer’s Disease: Translational Insights and Emerging Treatments

**DOI:** 10.3390/ph19040626

**Published:** 2026-04-15

**Authors:** Ivona Costachescu, Gabriela-Dumitrita Stanciu, Raluca Maria Gogu, Bogdan-Ionel Tamba

**Affiliations:** 1 Advanced Research and Development Center for Experimental Medicine “Prof. Ostin C. Mungiu”—CEMEX, Grigore T. Popa University of Medicine and Pharmacy Iasi, 700454 Iasi, Romaniabogdan.tamba@umfiasi.ro (B.-I.T.); 2 Department of Pharmacology, Clinical Pharmacology and Algesiology, Grigore T. Popa University of Medicine and Pharmacy Iasi, 700115 Iasi, Romania

**Keywords:** Alzheimer’s disease, pain, depression, comorbidities, pathophysiological mechanisms, neuroinflammation, oxidative stress, endocannabinoid system, translational research

## Abstract

Alzheimer’s disease (AD) is primarily recognized for progressive cognitive decline driven by beta-amyloid accumulation and tau pathology. However, many individuals with AD also experience chronic pain and depressive symptoms, which significantly impair daily functioning and quality of life and increase caregiver burden. These non-cognitive features are frequently underrecognized, despite evidence suggesting they share overlapping biological pathways with neurodegeneration. Emerging data highlight the role of neuroinflammation, oxidative stress, hypothalamic–pituitary–adrenal axis dysregulation, and endocannabinoid system alterations in linking AD pathology to disturbances in pain processing and mood regulation. Persistent microglial activation, cytokine imbalance, redox disruption, and chronic stress signaling may simultaneously promote neuronal vulnerability while shaping affective and nociceptive responses. This review synthesizes current preclinical and clinical evidence on the interplay between pain, depression, and AD, emphasizing their shared pathophysiological mechanisms and clinical relevance. Recognizing these symptoms as integral components of disease progression, rather than isolated comorbidities, can inform the development of integrated, multidimensional therapeutic strategies in AD care.

## 1. Introduction

Alzheimer’s disease (AD) is the most common neurodegenerative disorder worldwide, accounting for 60–70% of all dementia cases. More than 55 million people currently live with dementia, a number projected to exceed 139 million by 2050 due to global population aging [1]. AD is classically characterized by progressive cognitive decline, driven by beta-amyloid (Aβ) accumulation, tau pathology, synaptic and mitochondrial dysfunction, oxidative stress, and chronic neuroinflammation [2,3,4,5,6]. These pathological processes lead to neuronal loss and synaptic impairment, forming the biological basis of memory deficits and executive dysfunction [7,8,9]. Beyond cognitive decline, however, AD is increasingly recognized as a multifactorial disorder, in which non-cognitive symptoms contribute substantially to disease burden [10].

Among these, chronic pain and depressive disorders are particularly prevalent and clinically impactful. Epidemiological studies indicate that depression affects approximately 30–50% of patients with AD [11,12,13,14], while chronic pain is reported in 40–60% of cases, with higher prevalence in institutionalized populations [15,16,17]. Anxiety, sleep disturbances, and apathy are also common, often co-occurring with pain and depression and further impairing functional independence and quality of life [18,19,20,21]. These non-cognitive symptoms are associated with accelerated cognitive decline, increased behavioral disturbances, greater caregiver burden, and higher rates of hospitalization and institutionalization [18,19,20,21,22,23]. Despite their prevalence and clinical significance, pain and depression are frequently underrecognized and undertreated, partly due to diagnostic challenges and limited guidance from clinical trials that often exclude individuals with complex comorbidities.

Emerging evidence suggests that these non-cognitive comorbidities are not mere epiphenomena but share overlapping biological pathways with the mechanisms driving neurodegeneration. Neuroinflammation [24,25,26], oxidative stress [27,28,29,30], dysregulation of the hypothalamic–pituitary–adrenal (HPA) axis [31,32,33], persistent microglial activation [34,35,36,37,38,39], and alterations in the endocannabinoid system [14,16,40,41,42,43] have all been implicated in modulating affective states and pain perception while simultaneously promoting neuronal vulnerability. In this context, chronic pain and depression may not only be consequences of AD pathology but also modifiers of disease progression, contributing to synaptic dysfunction and network destabilization.

Understanding the interplay between cognitive and non-cognitive symptoms is therefore essential for developing integrated, multidimensional therapeutic strategies. Translational approaches that bridge preclinical findings with clinical interventions hold particular promise, enabling targeted modulation of the shared biological pathways underlying pain, depression, and neurodegeneration [44,45,46,47]. Recent advances in molecular and cellular research, combined with innovations in drug delivery and neurobehavioral assessment, provide opportunities to refine therapeutic strategies and improve outcomes for patients with AD.

This review aims to synthesize current preclinical and clinical evidence on pain and depression in AD, focusing on prevalence, pathophysiology, and emerging treatment strategies, with a particular emphasis on translational relevance and patient-centered care. By highlighting gaps in knowledge and potential therapeutic targets, we aim to provide a comprehensive framework for understanding these comorbidities as integral components of AD progression, rather than isolated phenomena.

## 2. Neurobiological and Clinical Perspectives on Pain–Depression Comorbidity in Alzheimer’s Disease

### 2.1. Clinical Impact on Disease Progression and Functional Decline

Pain and depression are complex and interrelated conditions with overlapping neurobiological mechanisms. The International Association for the Study of Pain (IASP) defines pain as “an unpleasant sensory and emotional experience associated with actual or potential tissue damage, or described in terms of such damage” [48]. This definition is particularly relevant in Alzheimer’s disease, where cognitive decline may interfere with the accurate perception, interpretation, and communication of pain experiences. Similarly, depression in AD encompasses a spectrum from subsyndromal mood disturbances to major depressive episodes and frequently overlaps with other neuropsychiatric symptoms such as apathy, anxiety, and irritability. Diagnostic challenges arise because cognitive deficits may mask classic affective symptoms or alter self-reporting. Recognizing pain and depression as multidimensional and interrelated syndromes is essential for understanding their clinical and mechanistic significance [44,45,46,47,49].

The clinical impact of pain–depression comorbidity in AD extends far beyond simple symptom frequency, affecting functional independence, cognition, behavior, and overall health. Patients with both chronic pain and depressive symptoms consistently show greater impairment in activities of daily living (ADLs), mobility, and executive functioning compared with patients affected by either condition alone or neither condition [50,51,52]. For example, in the Alzheimer’s Disease Neuroimaging Initiative (ADNI) cohort, baseline depressive symptoms were associated with a 1.5- to 2-fold acceleration in functional decline over 36 months, independent of baseline cognitive performance [53]. Population-based studies similarly indicate that AD patients with depression perform significantly worse on timed gait, balance, and chair-stand tests, with effect sizes comparable to those seen in individuals with more advanced cognitive impairment [54,55].

Behavioral and psychological symptoms of dementia (BPSD), including agitation, irritability, sleep disturbances, apathy, and nighttime wandering, are reported more frequently and with greater severity in patients experiencing both pain and depression [56,57]. These findings underscore the multidimensional burden of this comorbidity and its relevance for patient care. To provide a comprehensive overview, Table 1 summarizes representative clinical and preclinical studies investigating pain–depression comorbidity in AD, highlighting prevalence, behavioral and cognitive outcomes, and translational relevance.

Collectively, clinical and preclinical studies indicate that pain and depression in AD are interdependent and exert profound effects on cognition and behavior. Understanding the underlying neurobiological mechanisms is essential to explain these interactions and to guide the development of targeted therapeutic strategies.

### 2.2. Shared Pathophysiological Mechanisms, Integrative Perspective, and Translational Implications of Pain–Depression Comorbidity

**Figure 1:**
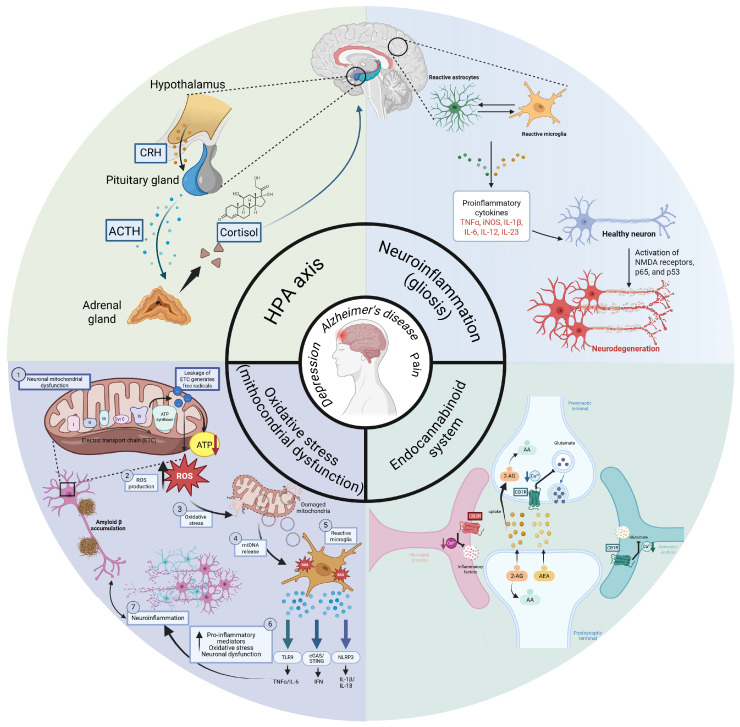
Overview of the main pathophysiological mechanisms common to Alzheimer’s disease, depression, and pain. The figure highlights four interconnected processes: dysregulation of the hypothalamic–pituitary–adrenal (HPA) axis, neuroinflammation, oxidative stress associated with mitochondrial dysfunction, and alterations of the endocannabinoid system. Amyloid-beta (Aβ) accumulation contributes to neuronal mitochondrial dysfunction, leading to increased production of reactive oxygen species (ROS) and oxidative stress. This, in turn, promotes microglial activation and the release of pro-inflammatory mediators, further amplifying neuroinflammatory signaling. Chronic activation of the HPA axis leads to increased cortisol levels, while glial reactivity contributes to cytokine release and neuronal damage [91]. Mitochondrial dysfunction increases reactive oxygen species (ROS) production and amplifies inflammatory signaling. In parallel, the endocannabinoid system modulates synaptic transmission, neuroinflammation, and pain signaling through CB1 and CB2 receptors. Together, these interacting mechanisms may contribute to the comorbidity observed between Alzheimer’s disease, depression, and chronic pain. The figure was created in BioRender. Gogu, R. (2026) https://BioRender.com/ienaoyv, (accessed on 8 April 2026). Abbreviations: CRH, corticotropin-releasing hormone; ACTH, adrenocorticotropic hormone; ATP, adenosine triphosphate; TNF-α, tumor necrosis factor alpha; iNOS, inducible nitric oxide synthase; IL-1β, interleukin-1 beta; IL-6, interleukin-6; IL-12, interleukin-12; IL-23, interleukin-23; NMDA receptors, N-Methyl-D-Aspartate receptors; 2-AG, 2-arachidonoylglycerol; AA, arachidonic acid; AEA, anandamide; NLRP3, NLR Family Pyrin Domain Containing 3; cGAS, cyclic GMP-AMP synthase; STING, stimulator of interferon genes; TLR9, Toll-like receptor 9; IL-18, interleukin-18; IFN, interferon; ETC, electron transport chain.

Pain and depression frequently co-occur in Alzheimer’s disease, yet the neurobiological mechanisms mediating this comorbidity remain incompletely defined. Evidence from clinical and preclinical studies indicates that these conditions converge on shared pathophysiological pathways, including neuroinflammation, oxidative stress, hypothalamic–pituitary–adrenal axis dysregulation, persistent microglial activation, and alterations in the endocannabinoid system that may collectively exacerbate cognitive and behavioral impairments and accelerate neurodegenerative processes (Figure 1).

Neuroinflammatory processes are central to both nociceptive modulation and mood regulation and are intricately linked to the core pathology of AD. Elevated levels of pro-inflammatory cytokines such as interleukin (IL)-1β, interleukin-6 (IL-6), and tumor necrosis factor-alpha (TNF-α) are observed in AD brains, and these mediators have been correlated with synaptic dysfunction and neurodegeneration [24,25,26,81,82]. Chronic pain activates peripheral and central immune responses, leading to sustained cytokine release and glial priming, which may in turn amplify depressive symptoms [83,84]. In preclinical AD models (e.g., APP/PS1, 5×FAD), peripheral inflammatory stimuli or chronic nociceptive input increase central cytokine expression, potentiate β-amyloid accumulation, and worsen depressive-like behaviors and memory deficits [74,75,76,77,85,86]. Clinically, elevated inflammatory markers in plasma and cerebrospinal fluid are associated with both pain severity and depressive symptoms in AD patients [87,88], supporting the translational relevance of this mechanism. Anti-inflammatory interventions, including microglial modulators (e.g., minocycline), have demonstrated efficacy in reducing pain sensitization and depressive phenotypes in animal models [89,90].

Oxidative stress reflects an imbalance between reactive oxygen species (ROS) generation and antioxidant defenses, contributing to neuronal damage and synaptic loss. In AD, oxidative damage correlates with amyloid and tau pathology and precedes overt cognitive decline [92,93,94]. Chronic pain states exacerbate ROS production and impair mitochondrial function in key brain regions such as the hippocampus and prefrontal cortex [95,96]. Preclinical studies report elevated markers of lipid peroxidation and decreased antioxidant enzyme activity in animals with chronic pain and depressive-like behavior [97]. Clinically, patients with AD and comorbid depression show increased oxidative biomarkers in blood and cerebrospinal fluid compared with non-depressed AD subjects [98,99]. Targeting oxidative stress with antioxidants (e.g., vitamin E, N-acetylcysteine) has been explored to ameliorate cognitive and affective deficits, though results are mixed and warrant further investigation [100,101,102].

The HPA axis coordinates the stress response, and its dysregulation features prominently in chronic pain, depression, and AD. Sustained stress and nociceptive input lead to prolonged glucocorticoid secretion, which exerts deleterious effects on hippocampal neurons and neurogenesis [103,104]. In rodent models, chronic stress or pain elevates corticosterone levels, reduces hippocampal synaptic density, and impairs memory performance [18,19]. In AD patients, alterations in diurnal cortisol rhythms and elevated basal cortisol levels have been associated with greater hippocampal atrophy and accelerated cognitive decline, particularly in those presenting with depressive symptoms [20,21]. Dysregulated HPA signaling may thus represent a shared mechanistic link through which chronic pain exacerbates mood disturbance and cognitive impairment, underscoring the potential utility of HPA modulators (e.g., glucocorticoid receptor antagonists) or structured stress-reduction interventions.

The endocannabinoid system (ECS) comprises two principal cannabinoid receptors (CB1R and CB2R), their endogenous ligands—anandamide (AEA) and 2-arachidonoylglycerol (2-AG)—and the enzymatic machinery responsible for their synthesis and degradation [41]. CB1 receptors are widely distributed throughout the central nervous system, particularly in limbic and stress-related regions such as the prefrontal cortex, hippocampus, amygdala, nucleus accumbens, and hypothalamus, whereas CB2 receptors are primarily expressed on immune cells, with central expression predominantly detected in microglia [41,105,106,107]. Through this neuroanatomical distribution, the ECS functions as a key neuromodulatory system integrating nociceptive processing, emotional regulation, cognitive function, and stress responsivity [108]. Endocannabinoid signaling also modulates HPA axis activity, thereby contributing to adaptive responses to stress and maintaining affective homeostasis [109]. These properties position the ECS at the intersection of biological pathways implicated in both pain perception and mood regulation. In the context of Alzheimer’s disease, growing evidence indicates that ECS dysregulation contributes to both neurodegenerative processes and neuropsychiatric manifestations [41,110]. Activation of cannabinoid receptors exerts anti-inflammatory and neuroprotective effects, partly through modulation of microglial activation and inflammatory signaling [111,112,113]. In particular, selective CB2 receptor stimulation has been shown to attenuate neuroinflammation, reduce the production of pro-inflammatory mediators, and facilitate amyloid-β clearance in experimental models and human-derived cellular systems [114,115]. Supporting these findings, the CB2R agonist MDA7 reduced Aβ1–40-induced neuroinflammation, synaptic dysfunction, and cognitive impairment in the hippocampus in preclinical studies [41]. Importantly, the ECS also plays a central role in descending pain modulation. CB1 receptor activation engages inhibitory pain circuits involving the amygdala, periaqueductal gray, and rostral ventromedial medulla. Accordingly, CB1R agonists such as Δ9-THC and WIN 55,212-2 have been shown to activate endogenous analgesic pathways [15,116]. Beyond nociception, ECS signaling critically influences neuroplasticity, stress adaptation, and affective regulation, processes frequently disrupted in depression [117]. Dysregulation of ECS tone has been associated with altered hippocampal neurogenesis and impaired stress responsivity, while pharmacological elevation of anandamide levels—through processes such as inhibition of fatty acid amide hydrolase (FAAH)—reduces depressive-like behaviors in preclinical models [118]. Consistently, clinical observations indicate that CB1R antagonism may precipitate mood disturbances, and reduced endocannabinoid levels have been reported in individuals with depressive disorders [119,120].

Taken together, the ECS represents a biologically plausible integrative interface linking neuroinflammation, HPA-axis dysregulation, nociceptive processing, and affective disturbances. Within the complex pathophysiology of AD, these mechanisms converge to shape the frequent comorbidity between pain and depression. Consequently, pharmacological modulation of these components may offer a translationally relevant therapeutic strategy capable of targeting multiple interacting pathways simultaneously, supporting its potential role in integrated treatment approaches for pain–depression comorbidity in Alzheimer’s disease.

## 3. Emerging Therapeutic Strategies

The frequent coexistence of pain and depression in Alzheimer’s disease reflects the convergence of multiple pathophysiological processes. Conventional pharmacological approaches, typically involving analgesics and antidepressants, often provide only partial symptom control and may be associated with adverse cognitive or systemic effects in elderly patients. Consequently, increasing attention has focused on emerging therapeutic strategies that target shared biological mechanisms underlying the pain–depression comorbidity.

### 3.1. Modulation of the Endocannabinoid System

Activation of cannabinoid receptors modulates nociceptive transmission within descending inhibitory pathways and influences emotional regulation via limbic circuits. Concurrently, ECS signaling regulates microglial activation and the release of inflammatory mediators, suggesting potential neuroprotective effects in Alzheimer’s disease [121]. Preclinical evidence demonstrates that cannabinoid-based compounds may produce both analgesic and antidepressant-like effects, highlighting their therapeutic potential for managing pain–depression comorbidity. Beyond direct receptor agonists, pharmacological approaches that enhance endogenous cannabinoid signaling, such as inhibition of endocannabinoid-degrading enzymes, are under investigation [116]. In rodent models of neuropathic pain, cannabinoid receptor agonists such as JWH-182 and WIN55,212-2 significantly reduced mechanical allodynia and thermal hyperalgesia through activation of descending inhibitory pathways involving the periaqueductal gray and rostral ventromedial medulla [115,122]. Similarly, pharmacological enhancement of endocannabinoid signaling through inhibition of FAAH increased anandamide levels and produced robust antidepressant-like effects in stress-induced rodent models [123]. Selective activation of CB2 receptors has been shown to attenuate microglial activation and reduce pro-inflammatory cytokine production in transgenic mouse models of Alzheimer’s disease [16,112]. For example, administration of CB2 receptor agonists in amyloid-β mouse models reduced neuroinflammation, improved synaptic function, and partially restored cognitive performance. In addition, ECS-targeting compounds have been reported to facilitate amyloid-β clearance by modulating microglial phagocytic activity, suggesting a potential disease-modifying role [124,125,126].

Clinical evidence supporting the therapeutic potential of ECS modulation has primarily emerged from studies in chronic pain and mood disorders. Cannabinoid-based medications have demonstrated analgesic efficacy in several clinical trials involving patients with neuropathic pain [127], multiple sclerosis-associated pain [128], and cancer-related pain [129]. In addition to analgesic effects, some clinical studies have reported improvements in sleep quality, anxiety, and mood symptoms following cannabinoid treatment [130,131].

Observational studies and small clinical trials have also explored the effects of cannabinoid-based therapies in patients with Alzheimer’s disease and other dementias [132,133,134]. In these populations, cannabinoids have been investigated primarily for the management of neuropsychiatric symptoms such as agitation, anxiety, and sleep disturbances. Although results remain heterogeneous and large-scale randomized trials are still limited, preliminary findings suggest that cannabinoid-based treatments may improve behavioral symptoms and overall patient comfort.

Collectively, these findings suggest that ECS modulation may represent a biologically plausible therapeutic approach capable of targeting multiple interconnected pathways implicated in the pain–depression interface. By simultaneously influencing nociceptive processing, neuroinflammatory signaling, and emotional regulation, ECS-targeting therapies may offer advantages over conventional treatments that focus on a single symptom domain. Nevertheless, further well-designed clinical trials are required to determine optimal dosing strategies, long-term safety profiles, and the potential cognitive effects of cannabinoid-based therapies in elderly patients with neurodegenerative disorders.

### 3.2. Anti-Inflammatory and Immune-Modulating Approaches

Preclinical studies have demonstrated that interventions capable of reducing microglial activation or inhibiting inflammasome pathways can ameliorate pain hypersensitivity and depressive-like behaviors in animal models of AD [135,136]. For example, minocycline and P2X7 receptor antagonists attenuate neuroinflammation [137,138,139,140], while NLRP3 inhibitors restore synaptic function and reduce behavioral deficits [136]. Similarly, cytokine-targeting approaches and strategies that enhance immune homeostasis—such as modulation of regulatory T cells or the endocannabinoid system—have shown dual benefits on nociceptive and affective outcomes, highlighting the intertwined nature of neuroinflammatory signaling in AD [141,142].

Clinically, anti-inflammatory interventions, including selective NSAIDs, cytokine inhibitors, and lifestyle-based strategies, have been explored for their potential to mitigate depressive symptoms and pain in patients with AD, particularly in those exhibiting elevated inflammatory biomarkers [143,144,145,146]. Although results remain preliminary, these findings suggest that targeting neuroinflammation may provide an integrated approach to managing both core and comorbid features of AD. Future research should focus on biomarker-guided patient stratification, CNS-penetrant agents, and co-therapies that address multiple inflammatory pathways, with the goal of translating preclinical mechanistic insights into meaningful clinical benefits.

### 3.3. Neuroplasticity-Enhancing Therapies

Preclinical studies provide compelling evidence that modulation of glutamatergic neurotransmission can rapidly reverse synaptic deficits and behavioral abnormalities across models of neurodegeneration, pain, and depression. NMDA receptor antagonists, most notably ketamine, have been shown to enhance synaptic spine density, restore long-term potentiation, and increase BDNF signaling in hippocampal and prefrontal circuits [147,148]. In animal models of AD, similar interventions not only improve cognitive performance but also attenuate depressive-like behaviors and reduce central sensitization associated with chronic pain. These effects are thought to involve downstream activation of mTOR-dependent pathways and normalization of cortico-limbic connectivity [149].

Clinical evidence, although still emerging, supports the relevance of these mechanisms in humans. Rapid-acting antidepressants targeting glutamatergic systems, such as ketamine and its derivatives, have demonstrated robust efficacy in treatment-resistant depression and are increasingly being explored for their potential to alleviate comorbid pain symptoms [150,151,152]. Early clinical studies also suggest that such agents may exert beneficial effects on cognitive and affective domains in patients with neurodegenerative disorders, although their long-term safety and disease-modifying potential in AD remain to be fully established. Additionally, interventions aimed at enhancing neurotrophic support—whether pharmacological or lifestyle-based, such as physical exercise—have been associated with improvements in mood, pain perception, and cognitive function, likely through activity-dependent increases in BDNF and synaptic plasticity [152,153].

Taken together, these findings highlight neuroplasticity as a central therapeutic target linking AD, chronic pain, and depression. Future research should prioritize the development of CNS-penetrant agents with sustained effects on synaptic remodeling, as well as biomarker-driven approaches to identify patients most likely to benefit from neuroplasticity-enhancing therapies. Integrating glutamatergic modulation with anti-inflammatory and neuroprotective strategies may further enhance clinical outcomes by addressing the multifactorial nature of neurodegenerative and neuropsychiatric comorbidities.

### 3.4. Neuromodulation Strategies

Non-pharmacological neuromodulation approaches have attracted growing interest as potential therapeutic tools for addressing the overlapping domains of pain and depressive symptoms in neurodegenerative disorders, including AD [154]. By directly modulating neuronal excitability within cortical and limbic circuits, particularly the prefrontal cortex, anterior cingulate cortex, and insular regions, techniques such as transcranial magnetic stimulation (TMS) and transcranial direct current stimulation (tDCS) may influence both nociceptive processing and mood regulation. In the context of AD, where network-level dysfunction and synaptic disconnection are prominent, these approaches offer a mechanistically distinct, circuit-based intervention strategy [155,156].

Preclinical evidence supports the capacity of neuromodulation to induce neuroplastic changes relevant to neurodegeneration and affective dysfunction. Experimental studies in animal models have shown that repetitive magnetic or electrical stimulation can enhance synaptic plasticity, increase neurotrophic factor expression (including BDNF), and modulate glutamatergic and GABAergic transmission [157,158,159,160,161]. These effects are associated with improvements in learning and memory, attenuation of pain-related hypersensitivity, and reduction in depressive-like behaviors [157,158,159,160,161,162,163]. Importantly, neuromodulation has also been linked to reduced neuroinflammatory signaling and improved network synchronization, suggesting convergence with other disease-modifying pathways implicated in AD [164].

Clinical studies further substantiate these findings, particularly in the domains of depression and chronic pain. Repetitive TMS has demonstrated robust efficacy in treatment-resistant depression, with additional evidence indicating beneficial effects on pain perception, especially in neuropathic and centralized pain syndromes [155,156]. Similarly, tDCS has shown modest but consistent effects in improving mood and reducing pain intensity, likely through sustained modulation of cortical excitability and functional connectivity. Neuroimaging studies in humans indicate that these interventions can normalize activity within disrupted cortico-limbic networks and influence neurotransmitter systems, including glutamate and monoamines [165,166].

In AD populations, clinical evidence remains comparatively limited but increasingly suggestive. Early-phase trials of TMS and tDCS have reported improvements in cognitive performance, with some studies also noting secondary benefits in mood and behavioral symptoms [167,168]. Although pain-specific outcomes are rarely assessed, the shared neural substrates between pain and affective processing raise the possibility of broader therapeutic effects. Notably, neuromodulation techniques are generally well tolerated, which may be particularly advantageous in elderly patients with polypharmacy and increased sensitivity to pharmacological interventions [167,168].

Collectively, neuromodulation represents a promising complementary strategy for targeting the network-level dysfunction that underlies cognitive decline, pain, and depression in AD. Future research should aim to optimize stimulation parameters, identify patient subgroups most likely to respond, and integrate neuromodulation with pharmacological and behavioral interventions. A multimodal approach that combines circuit-level modulation with anti-inflammatory and neuroplasticity-enhancing therapies may ultimately provide the greatest clinical benefit in this complex and heterogeneous condition.

### 3.5. Nanotechnology-Based Drug Delivery Systems

Preclinical studies provide substantial evidence supporting the utility of nanocarriers in overcoming BBB-related limitations. A wide range of nanosystems, including liposomes, polymeric nanoparticles, solid lipid nanoparticles, and dendrimers, have been engineered to improve drug solubility, stability, and bioavailability [169]. In animal models of AD, nanoparticle-based formulations have been used to deliver anti-inflammatory agents, antioxidants, and neuroprotective compounds directly to affected brain regions, resulting in reduced amyloid burden, attenuation of neuroinflammation, and improved cognitive performance [170,171,172]. Notably, nanocarriers have also been employed to enhance the central delivery of drugs targeting glutamatergic signaling and monoaminergic pathways, thereby influencing both pain processing and depressive-like behaviors. Functionalization of nanoparticles with targeting ligands (e.g., transferrin or insulin receptors) has further improved BBB transcytosis and cellular specificity, highlighting the potential for precision-based CNS delivery [173,174,175,176].

Clinical translation, although still at an early stage, is gradually advancing. Several nanoparticle-based formulations have entered clinical trials for CNS disorders, including liposomal and polymer-based systems designed to enhance drug delivery to the brain [177,178]. While most clinical studies in AD have focused on cognitive endpoints, emerging evidence indicates that improved CNS bioavailability may also translate into benefits for neuropsychiatric symptoms, including depression and potentially pain-related outcomes. However, significant challenges remain, including interspecies differences in BBB transport, large-scale manufacturing constraints, and long-term safety and regulatory considerations [179,180,181].

Taken together, nanotechnology-based delivery systems represent a rapidly evolving field with the potential to address key limitations in the treatment of AD and its comorbidities. By improving CNS penetration and enabling targeted, multi-drug delivery, these platforms may enhance therapeutic efficacy across cognitive, affective, and nociceptive domains. Future research should prioritize clinically translatable designs, biomarker-guided evaluation of CNS drug distribution, and the integration of nanotechnology with other emerging strategies, such as neuroinflammation modulation and neuroplasticity enhancement, to achieve more effective and personalized interventions.

### 3.6. Multi-Target and Precision Therapeutic Approaches

Preclinical studies provide strong support for the rationale of multi-target interventions [182]. In animal models of AD, combination therapies integrating anti-inflammatory agents, modulators of glutamatergic or monoaminergic signaling, and neuroprotective compounds have demonstrated synergistic effects on cognitive performance, pain sensitivity, and depressive-like behaviors [16,183,184,185,186]. Moreover, co-administration of anti-inflammatory drugs with NMDA receptor modulators or neurotrophic-enhancing agents has been shown to simultaneously reduce neuroinflammation, restore synaptic plasticity, and normalize behavioral outcomes [147]. Similarly, multifunctional compounds designed to act on several molecular targets within a single pharmacological entity have shown promise in attenuating amyloid pathology while also improving affective and nociceptive parameters [187]. These findings highlight the interconnected nature of the biological pathways involved and support a systems-level therapeutic approach.

Clinical research is beginning to reflect this paradigm shift. Combination therapies are increasingly explored in patients with AD and comorbid neuropsychiatric symptoms, often involving the adjunctive use of antidepressants, analgesics, and anti-inflammatory agents [188,189,190,191]. Although results remain heterogeneous, certain patient subgroups, particularly those with elevated inflammatory markers or pronounced neuropsychiatric symptoms, appear to derive greater benefit from such integrative approaches [192]. In parallel, clinical trials are investigating drugs with pleiotropic mechanisms of action, aiming to address multiple aspects of disease pathology within a single treatment regimen [193,194].

From a translational perspective, integrating multi-target strategies with precision medicine approaches represents a promising direction for improving outcomes in AD patients with pain–depression comorbidity. Future research should focus on validating robust biomarker panels, optimizing combination regimens, and establishing adaptive clinical trial designs that account for patient heterogeneity. By aligning therapeutic interventions with underlying pathophysiological mechanisms, these approaches may ultimately enhance efficacy, reduce adverse effects, and support the development of more personalized and clinically meaningful treatment strategies.

A summary of the main therapeutic strategies in Alzheimer’s disease with pain–depression comorbidity is presented in Table 2. The table integrates key preclinical and clinical findings, highlighting mechanisms of action, therapeutic targets, and translational potential.

## 4. Translational Perspectives and Future Directions

Bridging the gap between mechanistic insights and clinically effective interventions remains one of the central challenges in Alzheimer’s disease, particularly in the context of pain–depression comorbidity. Although substantial progress has been made in elucidating the roles of neuroinflammation, synaptic dysfunction, and large-scale network alterations, the translation of these findings into meaningful clinical outcomes has been hindered by the complexity and heterogeneity of the disease. Importantly, most current therapeutic strategies continue to focus on isolated targets, which may be insufficient to address the multifactorial nature of AD and its associated neuropsychiatric and nociceptive manifestations.

A critical priority for future research is the development of more predictive and integrative preclinical models. Traditional transgenic models of AD often fail to fully capture the interplay between neurodegeneration, chronic pain, and depressive-like behaviors, limiting their translational value [195,196]. Incorporating multimodal phenotyping encompassing behavioral, neurochemical, and neuroimaging details alongside models that reflect aging, comorbidities, and environmental stressors may provide a more accurate representation of human disease. Such approaches would facilitate the identification of convergent pathological pathways and improve the preclinical evaluation of multi-target therapeutic strategies.

Advances in biomarker discovery and neuroimaging are expected to play a transformative role in the transition toward precision medicine. The integration of peripheral inflammatory markers, cerebrospinal fluid biomarkers (including Aβ and tau), and advanced imaging modalities such as PET and functional MRI offers the potential to define biologically meaningful patient subgroups. This stratification is particularly relevant given the growing recognition that subsets of patients may exhibit inflammation-dominant, synaptic dysfunction-dominant, or mixed pathophysiological profiles [197,198]. Aligning therapeutic interventions with these profiles could enhance treatment efficacy and reduce variability in clinical trial outcomes.

From a therapeutic perspective, the convergence of multiple pathogenic mechanisms supports the development of multi-target and multimodal approaches. Strategies integrating anti-inflammatory agents, neuroplasticity-enhancing compounds, and neuromodulatory interventions may provide synergistic benefits across cognitive, affective, and nociceptive domains. In this context, neuromodulation techniques such as transcranial magnetic stimulation and transcranial direct current stimulation offer a non-invasive means of restoring network-level dysfunction, while nanotechnology-based drug delivery systems may overcome limitations related to blood–brain barrier permeability and enable targeted, sustained release of therapeutics. The combination of these approaches within a unified therapeutic framework represents a promising direction for future investigation.

However, several translational barriers remain. Interspecies differences in neurobiology, limited reproducibility of preclinical findings, and challenges in scaling complex therapeutic platforms all contribute to the high attrition rate in clinical development [179,180,181]. Furthermore, the design of clinical trials in AD must evolve to incorporate biomarker-guided inclusion criteria, adaptive trial designs, and endpoints that extend beyond cognitive measures to include pain and depressive symptoms. Longitudinal studies assessing the durability of treatment effects and real-world functional outcomes will also be essential for establishing clinical relevance.

The integration of pharmacological treatments with technological and lifestyle-based interventions such as neuromodulation, digital health tools, and behavioral therapies may further enhance therapeutic outcomes. Ultimately, advancing toward a precision medicine framework that aligns treatment selection with individual biological signatures holds the greatest promise for improving quality of life and reducing the overall disease burden in patients with AD.

## Figures and Tables

**Table 1:** Representative clinical and preclinical studies supporting pain–depression comorbidity in Alzheimer’s disease.

Study Type	Population/Model	Measure/Assessment	Key Findings and Impact
Clinical study [58]	479 dementia referrals, aged-care residents	PainChek^®^ for pain, NPI for BPSD	65.6% had pain; 48.4% moderate–severe pain; patients in pain 3.8× more likely to show agitation/aggression. Strong association with BPSD severity and caregiver distress.
Clinical study [59]	52 verbally communicative nursing home residents; 40% with dementia	Structured pain interview; 0–10 numeric rating scale	residents with dementia reported higher pain intensity (median 8 vs. 6) despite similar chronic pain diagnoses; residents without dementia more likely to have opioid prescription (OR 4.37, *p* = 0.018)
Clinical study [60]	96 nursing home residents with moderate–severe cognitive impairment	APS for pain; CMAI for agitation	analgesics used by 34% (33/96); positive correlation between APS and CMAI (r = 0.45, *p* < 0.0001); subjects with higher agitation received more sedatives, but higher pain scores did not correlate with increased analgesic treatment
Clinical study [61]	16,836 community-dwelling adults ≥50 years; categorized as dementia, CIND, or intact cognition	pain presence, severity, interference; over-the-counter pain medications and opioids	dementia was linked to lower pain presence (OR = 0.61), interference (OR = 0.46), severity, and treatment use (OTC: 0.60; opioids: 0.33; prescriptions: 0.46), with CIND showing moderate reductions in pain and OTC use
Clinical study [62]	202 community-dwelling older adults with mild-to-moderate dementia and moderate-to-severe pain	questionnaires on predisposing (age, gender, race, education, care partner), enabling (income) and need factors (pain interference, depressive symptoms, cognition)	higher income and greater pain interference predicted analgesic use (OR = 0.79 each); low pain interference and low depressive symptoms predicted no treatment (b = −0.52, *p* = 0.005)
Clinical study [63]	320 older adults: 146 AD, 174 healthy controls;	neuropsychological assessments; pain: frequency, intensity and severity; depression: validated scales, social determinants of health measures	depression mediated the pain–AD link; chronic pain → depression (*p* = 0.050) → AD (*p* = 0.001); high R^2^; adverse social determinants linked to more pain and depression
Clinical study [64]	patient–caregiver dyads; community-dwelling persons with dementia	patient self-report (“pain right now”); caregiver proxy report; agitation assessment; multivariate dyadic congruence analysis	32% of patients self-reported current pain vs. 52% caregiver-reported; 59% dyadic agreement. Congruence increased if the patient was male (OR = 3.7) and decreased with higher agitation (OR = 0.94).
Clinical study [65]	7609 community-dwelling Medicare beneficiaries ≥65 years; 802 with dementia	modified validated dementia algorithm; self/proxy report of bothersome pain and activity-limiting pain; multivariable Poisson regression	63.5% of dementia participants reported bothersome pain, 43.3% activity-limiting; 30.3% rarely/never used meds; proxies reported slightly more pain; pain linked to comorbidities, low education, ADL disability, and depression/anxiety
Clinical study [66]	688 healthy community-dwelling older adults; 10-year follow-up	pain: MOS SF-36; cognition: processing speed, attention, memory, reasoning, global cognitive status; multilevel longitudinal models	baseline pain (31%) → worse memory, faster processing decline; incident pain (42%) → faster decline in multiple cognitive domains; persistent pain → accelerated memory loss
Clinical study [67]	8835 adults ≥45 years; cognitive trajectories classified as stable vs. rapid decline	number of pain sites; depressive symptoms (validated scale); logistic regression and mediation analysis	multi-site pain associated with rapid cognitive decline (adjusted OR = 1.30, 95% CI: 1.14–1.48); depressive symptoms predicted rapid decline (adjusted OR = 1.49, 95% CI: 1.32–1.68). Depression mediated 25.7% of the total effect of pain on cognitive decline.
Clinical study [68]	6983 adults ≥50 years; 16-year follow-up	pain trajectories (growth mixture modeling); cognitive function measures; neighborhood social cohesion and physical disorder	persistent moderate/severe pain → lower cognition vs. mild pain; neighborhood factors largely non-significant, though high social cohesion and low disorder improved cognition in the mild pain group
Clinical study [69]	153 nursing home residents with dementia	4 self-report tools; 3 observational scales; 2 informant questionnaires	only 60% could complete ≥1 self-report tool. Strongest correlations observed within same assessment type (self-report with self-report, observational with observational, informant with informant). Between-type agreement was substantially lower.
Clinical study [70]	352 patients with advanced dementia	MOBID-2, ICC, SPTP	MOBID-2: good test–retest reliability (ICC 0.81–0.85), responsive to treatment; moderately correlated with agitation (r = 0.35), not ADL; detects true pain changes, links pain to agitation
Clinical study [71]	25 patients with moderate–severe dementia	DCM and CMAI	acetaminophen increased social interaction and engagement, reduced isolation; scheduled analgesia may boost function and social activity in advanced dementia, even without reducing agitation
Clinical study [72]	199 nursing home residents; various dementia stages	MOBID-2, self-report scales, dementia subtype, medication review	pain prevalence 43% (MOBID-2), one-third of residents with pain had moderate–severe pain despite scheduled analgesics. Severe dementia associated with higher observed pain (27% vs. 15%). Self-reported pain higher in vascular dementia (54%) vs. AD (18%). Nociceptive pain predominant (72%). Acetaminophen most prescribed (80%).
Clinical study [73]	1114 community-dwelling adults ≥70 years; mean follow-up 4.4 years	pain intensity and interference, incident dementia (DSM-IV)	114 developed dementia. Pain intensity not associated with incident dementia. Higher pain interference significantly associated with increased dementia risk. When both included in the model, only pain interference remained significant.
Preclinical study [74]	5×FAD mice vs. wild-type	mechanical allodynia testing	5×FAD mice showed reduced mechanical allodynia; supports central neurodegenerative mechanisms underlying altered pain perception in AD
Preclinical study [75]	APP/PS1 mouse model	Pain threshold (von Frey); cognition (NOR, Morris water maze, Y-maze, passive avoidance); depression-like tests;	reduced pain threshold; worsened learning/memory deficits; increased depression-like behaviors.
Preclinical study [45]	APP/PS1 mice with neuropathic pain induced by SNI	Von Frey (mechanical threshold); Morris water maze (spatial memory)	neuropathic pain worsened learning and memory deficits.
Preclinical study [76]	Chronic monoarthritis in APP/PS1 mice	Morris Water Maze Task, spontaneous pain (flinching) and stimulus-evoked pain (limb use)	chronic pain accelerated cognitive impairment in APP/PS1 mice
Preclinical study [77]	APP/PS1 mice	EPM, NOR, left–right discrimination learning, tail suspension test, Habituation–dishabituation test	depressive/anxiety-like behaviors and memory impairments appear before β-amyloid plaques; shared genetic signatures suggest late-life depression may signal early AD
Preclinical study [78]	mice with chronic neuropathic pain → depression-like behaviors	depression-like behaviors, mechanical allodynia	chronic pain inhibits AHN via CXCR2 downregulation; CXCR2 overexpression restores AHN and reduces depression-like behaviors—potential therapeutic target
Preclinical study [79]	hTau transgenic mice—chronic restraint stress to induce depressive-like behaviors and cognitive decline	OFT, SPT, FST, NOR	chronic restraint stress increased depressive-like behaviors and cognitive deficits
Preclinical study [80]	5×FAD mice	OFT, SPT, FST	early depressive-like behaviors observed before cognitive deficits

BPSD, behavioral and psychological symptoms of dementia; NPI, Neuropsychiatric Inventory; APS, Abbey Pain Scale; CMAI, Cohen–Mansfield Agitation Inventory; CIND, cognitive impairment, no dementia; ADL, activities of daily living; MOS SF-36, Medical Outcomes Survey SF-36-Item; MOBID-2, Mobilization–Observation–Behavior–Intensity–Dementia-2; ICC, test–retest reliability; SPTP, responsiveness to stepwise pain treatment protocol; DCM, Dementia Care Mapping; NOR, Novel Object Recognition Test; SNI, sciatic nerve injury; EPM, elevated plus maze; ANH, adult hippocampal neurogenesis; CXCR2, C-X-C motif chemokine receptor 2; OFT, open-field test; SPT, sucrose preference test; FST, forced swim test.

**Table 2:** Emerging therapeutic strategies targeting pain–depression comorbidity in Alzheimer’s disease.

Therapeutic Strategy	Mechanism of Action	Representative Animal Studies	Representative Clinical Evidence	Translational Relevance
Endocannabinoid modulation	CB1/CB2 receptors, endocannabinoid enzymes	CB2 receptor agonists reduce neuroinflammation and cognitive impairment in Aβ-induced mouse models; cannabinoid agonists attenuate neuropathic pain and depressive-like behaviors in rodents [16,112,122,123,124]	Cannabinoid-based medicines show analgesic benefits in chronic pain patients and mood-modulating effects in depressive disorders [127,128,129,130,131,132,133,134]	ECS modulation may simultaneously influence nociceptive processing, neuroinflammation, and emotional regulation
Anti-inflammatory therapies	inhibition of microglial activation and cytokine signaling	Inhibition of inflammasome signaling reduces neuroinflammation and behavioral deficits in AD mouse models; anti-inflammatory interventions attenuate pain hypersensitivity in rodents [137,138,139,140]	Clinical studies suggest anti-inflammatory agents may improve depressive symptoms in inflammatory conditions and reduce pain in certain chronic disorders [141,142,143,144,145,146]	Targeting neuroinflammation may address shared biological mechanisms underlying pain and depression
Neuroplasticity-enhancing agents	restoration of synaptic plasticity and neurotrophic signaling	Glutamatergic modulators restore synaptic plasticity and reduce depressive-like behavior in rodent stress models [147,148,149]	Rapid-acting antidepressant therapies have demonstrated significant efficacy in treatment-resistant depression [150,151,152,153]	Restoration of synaptic plasticity may normalize neural circuits involved in both pain processing and affective regulation
Neuromodulation techniques	modulation of cortical and limbic circuit activity	Experimental stimulation paradigms in animals modulate pain perception and stress-related behaviors [157,158,159,160,161,162,163,164]	Transcranial magnetic stimulation shows clinical efficacy in major depressive disorder and chronic pain syndromes [165,166,167,168]	Neuromodulation may directly influence cortico-limbic networks implicated in pain and mood
Nanotechnology-based delivery	improved drug transport across the blood–brain barrier	Nanoparticle formulations enhance brain delivery of anti-inflammatory and neuroprotective compounds in experimental models [169,170,171,172,173,174,175,176]	Early clinical translation of nanomedicine approaches targeting CNS disorders [177,178,179,180,181]	Improved drug delivery across the blood–brain barrier may enhance therapeutic efficacy
Multi-target therapies	simultaneous modulation of multiple biological pathways	Combination therapies targeting inflammation and neurotransmission improve behavioral outcomes in rodent models [16,183,184,185,186]	Increasing clinical interest in multi-modal treatment strategies for complex neuropsychiatric conditions [188,189,190,191,192,193,194]	Integrated approaches may better address the multifactorial pathophysiology of pain–depression comorbidity

## Data Availability

No new data were created or analyzed in this study. Data sharing is not applicable to this article.

## Abbreviations

The following abbreviations are used in this manuscript:

AD	Alzheimer’s disease
Aβ	Beta-amyloid
HPA	Hypothalamic–pituitary–adrenal axis
ADLs	Activities of daily living
ADNI	Alzheimer’s Disease Neuroimaging Initiative
BPSD	Behavioral and psychological symptoms of dementia
NPI	Neuropsychiatric Inventory
IASP	International Association for the Study of Pain
APS	Abbey Pain Scale
CMAI	Cohen–Mansfield Agitation Inventory
CIND	Cognitive impairment, no dementia
MOS SF-36	Medical Outcomes Survey SF-36-Item
MOBID-2	Mobilization–Observation–Behavior–Intensity–Dementia-2
ICC	Test–retest reliability
SPTP	Responsiveness to stepwise pain treatment protocol
DCM	Dementia Care Mapping
NOR	Novel Object Recognition Test
SNI	Sciatic nerve injury
EPM	Elevated plus maze
ANH	Adult hippocampal neurogenesis
CXCR2	C-X-C motif chemokine receptor 2
OFT	Open-field test
SPT	Sucrose preference test
FST	Forced swim test
IL-1β	Interleukin-1β
TNF-α	Tumor necrosis factor-alpha
ROS	Reactive oxygen species
ECS	Endocannabinoid system
AEA	Anandamide
2-AG	2-Arachidonoylglycerol
FAAH	Fatty acid amide hydrolase
CRH	Corticotropin-releasing hormone
ACTH	Adrenocorticotropic hormone
ATP	Adenosine triphosphate
iNOS	Inducible nitric oxide synthase
AA	Arachidonic acid
NDMA receptors	N-Methyl-D-Aspartate receptors
NLRP3	NLR Family Pyrin Domain Containing 3
cGAS	Cyclic GMP-AMP synthase
STING	Stimulator of interferon genes
TLR9	Toll-like receptor 9
IFN	Interferon
TMS	Transcranial magnetic stimulation
tDCS	Transcranial direct current stimulation
